# Nitric Oxide-Releasing Thermoresponsive Pluronic F127/Alginate Hydrogel for Enhanced Antibacterial Activity and Accelerated Healing of Infected Wounds

**DOI:** 10.3390/pharmaceutics12100926

**Published:** 2020-09-28

**Authors:** Jiafu Cao, Mingzhi Su, Nurhasni Hasan, Juho Lee, Dongmin Kwak, Dong Young Kim, Keonwoo Kim, Eun Hee Lee, Jee H. Jung, Jin-Wook Yoo

**Affiliations:** 1 College of Pharmacy, Pusan National University, Busan 46241, Korea; caojiafu1985@163.com (J.C.); smz0310@163.com (M.S.); hasni1986.nh@gmail.com (N.H.); jhlee2350@gmail.com (J.L.); kdm318@naver.com (D.K.); jhjung@pusan.ac.kr (J.H.J.); 2 College of Pharmacy, Korea University, Sejong 30019, Korea; kkoolbam@naver.com (D.Y.K.); vipy2k96@naver.com (K.K.); eunheelee@korea.ac.kr (E.H.L.)

**Keywords:** thermoresponsive, pluronic F127, S-nitrosoglutathione, infected wound healing, antibacterial

## Abstract

Nitric oxide (NO), a highly reactive and lipophilic molecule, is one of the molecules present in the wound environment and implicated as an important regulator in all phases of wound healing. Here, we developed an NO-releasing thermoresponsive hydrogel (GSNO-PL/AL) composed of S-nitrosoglutathione (GSNO), pluronic F127 (PL), and alginate (AL) for the treatment of infected wounds. The GSNO was incorporated into the thermoresponsive PL/AL hydrogel, and differential scanning calorimetry techniques were used for the hydrogel characterization. The hydrogel was assessed by in vitro NO release, antibacterial activity, cytotoxicity, and wound-healing activity. The GSNO-PL/AL hydrogel demonstrated thermal responsiveness and biocompatibility, and it showed sustained NO release for 7 days. It also exhibited potent bactericidal activity against Gram-positive methicillin-resistant *Staphylococcus aureus* and Gram-negative multidrug-resistant *Pseudomonas aeruginosa* (MRPA). Moreover, the GSNO-PL/AL treatment of MRPA-infected wounds accelerated healing with a reduced bacterial burden in the wounds. The GSNO-PL/AL hydrogel would be a promising option for the treatment of infected wounds.

## 1. Introduction

Nitric oxide (NO), a diatomic free radical, is a cell-signaling molecule that is constitutively expressed in endothelial cells through the enzymatic conversion of L-arginine by nitric oxide synthase (NOS) [1]. The physiological roles of NO include neurotransmission, wound healing, blood pressure regulation, platelet adhesion, and immune response [2,3,4,5,6,7]. Likewise, NO also has strong efficacy against a wide range of Gram-positive and Gram-negative bacteria and plays a vital role in wound healing by modulating cell proliferation, wound contraction, and collagen deposition [8,9,10].

Despite the beneficial properties of NO, its utilization as an effective therapy is limited due to its gaseous property and short half-life (3–4 s). Researchers have focused on the development of exogenous NO donors [11,12]. S-nitrosoglutathione (GSNO), an endogenous NO carrier, has been used in experimental medical applications as an exogenous NO donor [13]. Nevertheless, the limitation of the GSNO application is its fast and often unpredictable rate of decomposition in aqueous solutions [14]. This decomposition of GSNO to release NO is mediated through the cleavage of the S−NO bond, which can occur through light and thermal decomposition, as well as metal ion particle catalysis [15]. Therefore, the development of the dosage forms to protect GSNO from environmental decomposition is necessary for further applications.

Hence, much research has been focused on the development of GSNO delivery systems, such as hydrogel, ointment, film, and micro/nanoparticles, in which it is used as an antimicrobial agent and a mediator of wound repair [16,17,18,19,20]. It is well known that maintaining a moist wound environment facilitates the wound-healing process [21,22]. Moreover, research has shown that moist wound healing is three to five times quicker than dry wound healing [23]. Wound dressings that create and maintain a moist environment are now considered to provide the optimal conditions for wound healing [24,25]. Among different wound dressers widely used in wound healing, hydrogels hold great promise because of their special properties [26,27].

This study investigated a pluronic F127 (PL)-based thermoresponsive hydrogel formulation, a liquid form at low temperature that becomes a gel at body temperature, for the local delivery of GSNO to cutaneous wounds [28]. The thermoresponsive hydrogel has an advantage over other GSNO-releasing hydrogels on GSNO stability, easy application, and controlled GSNO release. Considering that GSNO degrades faster as a temperature increases and is thermally instable, PL-based thermoresponsive hydrogel can minimize drug loss during the fabrication process because GSNO is incorporated into an aqueous PL solution at low temperatures. It also facilitates an easy application on different shapes of wound surfaces because the thermosensitive hydrogel has fluidity at low temperatures. Furthermore, the formation of a gel from a liquid at body temperature can maintain a moisture condition at a wound site, which can accelerate the wound healing process. PL, a biocompatible thermosensitive triblock copolymer hydrogel that is composed of a central hydrophobic mixture of polypropylene glycol and two hydrophilic polylactic acid blocks (PEO-PPO-PEO), is one of the very few synthetic polymeric materials approved by the U.S. Food and Drug Administration (FDA) for use in clinical applications [29]. PL has been reported to be well-tolerated and useful in topical, preventive, and ophthalmic preparations without skin irritation or sensitivity [30,31]. However, PL hydrogel has some undesirable characteristics such as weak mechanical strength, rapid erosion, and toxicity. The toxicity of PL formulation is related to the copolymer (PEO–PPO–PEO) concentration. To address this problem, the addition of macromolecules to PL hydrogel has been proposed for the construction of composite hydrogels to improve their properties [32]. Among various macromolecules, alginate (AL) (a natural polysaccharide extracted from brown sea algae) in PL has shown significant improvements in cell viability [33,34].

Therefore, we developed NO-releasing thermoresponsive pluronic F127/alginate hydrogel (GSNO-PL/AL) for the effective treatment of infected wounds using PL and AL as the thermoresponsive polymer and thickening agent, respectively. The GSNO-PL/AL was characterized by gelation temperature, differential scanning calorimetry (DSC), and in vitro release studies. The in vitro antibacterial activity of the GSNO-PL/AL was assessed against methicillin-resistant *Staphylococcus aureus* (MRSA) and multidrug-resistant *Pseudomonas aeruginosa* (MRPA), while a cytotoxicity test was also performed in L929 cells. Finally, the therapeutic efficacy of the GSNO-PL/AL was evaluated in an MRPA-infected wound model in the imprinting control region (ICR) mice.

## 2. Materials and Methods

### 2.1. Materials

Pluronic F127 (PL), alginate (AL), sodium nitrite, Mayer’s hematoxylin, eosin-Y disodium salt, 2,2,2-tribromoethanol, tert-amyl alcohol (2-methyl-2-butanol) (Avertin anesthesia component), and thiazolyl blue tetrazolium bromide (MTT) were purchased from Sigma-Aldrich (St. Louis, MO, USA). Glutathione (GSH) was purchased from Wako Pure Chemical (Osaka, Japan). Methicillin-resistant *Staphylococcus aureus* 3089 (MRSA) and multidrug-resistant *Pseudomonas aeruginosa* 2200 (MRPA) were purchased from the Korea National Research Resource Bank (KNRRB, Seoul, Korea). BactoTM tryptic soy broth (TSB) and DifcoTM Luria Bertani (LB) media were purchased from BD Biosciences (Sparks, MD, USA). Dulbecco’s modified Eagle medium (DMEM), fetal bovine serum (FBS), and penicillin–streptomycin were purchased from Hyclone (Thermo Fisher Scientifc Inc., Waltham, MA, USA). Masson’s trichrome stain (connective tissue stain) was purchased from Abcam (Cambridge, MA, USA). All other reagents and solvents were of analytical grade.

### 2.2. GSNO Synthesis

The synthesis of GSNO by reacting GSH with sodium nitrite (NaNO_2_) follows the previously reported method with some modifications [20]. GSH and NaNO_2_ were added to a HCl solution in an ice bath. The final concentration of GSH, NaNO_2_, and HCl was 0.625 M. After stirring for 40 min, cold acetone was added to precipitate the GSNO. The precipitate was collected by filtration and washed once with 80% acetone, twice with 100% acetone, and thrice with diethyl ether. After drying, the pink solid GSNO was stored in a −20 °C refrigerator for subsequent experiments.

### 2.3. Preparation of PL/AL Hydrogel

The cold method was employed to prepare PL/AL hydrogel at 4 °C. Firstly, AL solution was prepared by dissolving in distilled water under magnetic stirring at room temperature (25 ± 3 °C). Secondly, PL was gradually introduced to the AL solution. Then, it was kept at 4 °C overnight to make the PL powder dissolute completely, and final concentrations of PL and AL in the hydrogel were 20% *w*/*v* and 1% *w*/*v*, respectively.

### 2.4. Incorporation of GSNO into PL/AL Hydrogel

GSNO was incorporated into the PL/AL hydrogel matrix following the reported method with minor modification [35]. Two g of the GSNO powder was added to 100 mL of the PL/AL solution under gentle magnetic stirring and avoiding light at 4 °C. This process led to the formation of a GSNO-PL/AL hydrogel, and then, the final product was stored at 4 °C.

### 2.5. Measurement of Gelation Temperature

Gelation, the temperature at which the meniscus would no longer move upon tilting through 90 °C, was evaluated according to the method described by Tirnaksiz and Robinson (2005) [36]. Aliquots (1 mL) of the prepared formulation were sealed and transferred in test tubes to the water bath at 4 °C. Furthermore, the temperature was increased 2 °C per step until 20 °C and then with a final 0.2 °C increment.

### 2.6. Rheological Analyses of Hydrogels

Changes of the rheological properties of PL/AL and GSNO-PL/AL hydrogels with the temperature were investigated using a Rotational strain-controlled rheometer (Advanced Rheometric Expansion System (ARES), Rheometric Scientific, Piscataway, NJ, USA) equipped with a parallel-plate fixture with a radius of 25 mm an angular frequency of 1.0 rad/s, and a gap size of 0.5 mm. The temperature sweep analysis was performed in the range of 5–40 °C with a heating rate of 1 °C/min. Before experiment initiation, samples were rested for 10 min after loading on the plate for stabilization.

### 2.7. Thermal Analyses of Hydrogels

The differential scanning calorimetry (DSC) technique was used to determine the thermal characterization of the PL, PL/AL, and GSNO-PL/AL hydrogels using the DSC Q200 equipment (TA Instruments, New Castle, DE, USA). Samples of 5 mg of PL, PL/AL, and GSNO-PL/AL hydrogels were analyzed in the range of 0–50 °C with a heating rate of 1 °C/min, using nitrogen to purge the gas chamber (50 mL/min). The data were analyzed by Universal Analysis 2000 software (TA Instruments version 4.5 A, New Castle, DE, USA).

### 2.8. In Vitro NO Release

Real-time NO release from the GSNO-PL/AL hydrogel was detected by chemiluminescence in a nitric oxide analyzer (Sievers 280i NOA; GE Analytical Instruments Inc., Boulder, CO, USA). The chemiluminescence was measured to evaluate NO release kinetics at 37 °C under argon (Ar) gas flow. The GSNO-PL/AL hydrogel (500 mg) was used to analyze the NO release profile, and the maximum instantaneous NO release concentration [NO]_max_, the time required to reach the NO_max_ (t_m_), the NO release half-life (t_1/2_), and the duration of NO release (t_d_) were evaluated from the NO release profile.

### 2.9. Cytotoxicity of Hydrogels

The cytotoxicity of the PL/AL and GSNO-PL/AL hydrogels were evaluated in L929 mouse fibroblasts (KCLB, Seoul, Korea) by the tetrazolium-based colorimetric (MTT) assay. The L929 cells were cultured in DMEM supplemented with 10% (*v/v*) FBS and antibiotics (100 IU/mL of penicillin G sodium and 100 µg/mL of streptomycin sulfate). The cells were maintained in an incubator supplied with 5% CO_2_ air humidified atmosphere at 37 °C. Cells were seeded in 48-well plates and incubated for 24 h. Then, the culture medium was replaced with fresh media containing the hydrogels with different concentrations (2, 10, 20, 100, and 200 mg/mL) and incubated for 24 h. A standard MTT solution (5 mg/mL) in phosphate buffered saline (PBS) was added to each of the wells and incubated for 2 h. After removing the MTT solution, 300 μL of DMSO was added to each well, and the absorbance measured at 540 nm was proportional to the concentration of viable cells in each well. The data were expressed as mean ± standard deviation (SD) of eight replicates. The cell viability was calculated using the following Equation (1).

Cell viability (%) = (Absorbance (treated cells))/(Absorbance (control cells)) × 100(1)

### 2.10. In Vitro Antibacterial Activity

The antibacterial activity of GSNO, PL/AL, and GSNO-PL/AL hydrogels against MRPA and MRSA was evaluated by enumerating the colony-forming units (CFU) and modified Oxford cup method. The MRSA and MRPA were grown in LB and TSB media, respectively, at 37 °C and harvested in the mid-exponential growth phase. The bacterial pellets were re-suspended at a density of 10^8^ CFU/mL in PBS for the in vitro antibacterial assay. The hydrogels with different concentrations were added to 48-well plates, and 100 μL of the bacterial suspension was added dropwise to each sample. After 24 h incubation, the bacterial solution was centrifuged at 4000 rpm for 10 min and re-suspended in 1 mL of PBS. The loss of bacterial viability was evaluated by the colony-counting method. Briefly, ten-fold dilutions (100 μL each) of MRSA and MRPA were spread onto LB and TSB agar plates, respectively, and incubated overnight at 37 °C. Then, the colonies on each plate were counted to confirm bacterial viability.

For macroscopic antibacterial activities, the bacterial suspension (100 μL) was spread onto an LB and TSB agar plate and allowed to air dry for 10 min. Medical-grade silicone O-rings (inner diameter, 10 mm; outer diameter, 19 mm; thickness, 3 mm) were placed on top of the inoculated LB agar plate. The hydrogels and GSNO solution (30 μL) were added inside the silicone O-ring and incubated at 37 °C for 24 h.

### 2.11. In Vivo Wound-Healing Activity

All the animal experiments were reviewed and approved by the Pusan National University Institutional Animal Care and Use Committee (PNU-IACUC) on 1 February 2018 (Approval No. PNU-2018-1800). ICR mice (7–8 weeks old) weighing 30 ± 2 g were purchased from Samtako Bio Korea (Osan, Korea). The animals were maintained in the university animal center at room temperature under a 12 h light/12 h dark cycle. Full-thickness bacteria-infected burn wounds were created using the protocol reported by Yergoz et al. [37], with modifications. The mice were anesthetized with avertin before dorsal wound induction, and their mid-dorsal hair was shaved with an electric razor. Then, 8-mm diameter full-thickness wounds were induced with a punch. The wounds were cleaned with normal saline and disinfected with 75% (*v/v*) ethanol. Further, 20 µL of 6 × 10^9^ CFU/mL MRPA suspension was inoculated in the wound site to induce infection. The mice were randomly divided into the PL/AL hydrogel, GSNO-PL/AL hydrogel, and untreated groups. After 1 day, PL/AL and GSNO-PL/AL (20 µL) were topically applied to the test wounds, which were then covered with 3M™ Tegaderm™ film, secured with gauze, and fixed with adhesive tape. The wounds were photographed on days 1, 3, 5, 7, 9, and 11 after injury, and the wound areas were measured with the ImageJ software (1.48v, National Institutes of Health, Bethesda, MD, USA). Then, the wound size reduction rates were calculated as follows (Equation (2)):

Wound size reduction (%) = W_t_/W_0_ × 100 (2)

where W_0_ is the wound area on the day of surgery, and W_t_ is the wound area at the specified time.

### 2.12. Histological Analysis

Cross-sections of the full-thickness skin specimens were collected on the last day of the experiment. Skin samples were fixed for 24 h in 10% (*v/v*) formaldehyde and embedded in FSC 22 Frozen Section Compound (Leica Microsystems, Wetzlar, Germany). Vertical sections of 5 μm thickness were fixed onto glass slides and stained with hematoxylin and eosin (H & E) and the Masson’s trichrome stain to observe morphology and collagen formation, respectively. The slides were examined by light microscopy (Olympus BX53; Olympus Corp., Tokyo, Japan), and the images were digitally captured at a 1360 × 1024 pixel resolution with an Olympus DP70 digital camera (Olympus Corp., Tokyo, Japan).

### 2.13. Reduction of Bacterial Burden in Wounds

The wound sites of representative mice were sampled at 11 days after surgery to quantify the MRPA. Each sample was minced and homogenized in 1 mL of PBS. Then, 100 μL of the wound homogenate was serially diluted (1:10), plated on cetrimide-agar media, and incubated overnight. The CFU were determined by counting the colonies.

### 2.14. Statistical Analysis

The data were analyzed by a paired t-test in SigmaPlot v. 12.0 (SYSTAT Inc., Chicago, IL, USA) and expressed as mean ± standard deviation (SD). Statistical significance was set at *p* < 0.05.

## 3. Results and Discussion

### 3.1. Synthesis and Characterization of GSNO-PL/AL Hydrogel

**Figure 1:**
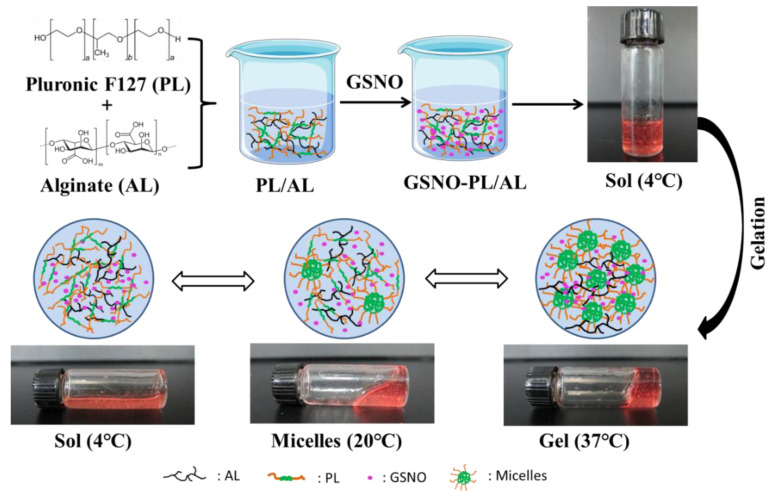
Schematic diagram of GSNO-PL/AL hydrogel synthesis and sol−gel transition. AL: alginate, GSNO: S-nitrosoglutathione, PL: pluronic F127.

In this study, GSNO-PL/AL hydrogel was synthesized by the incorporation of GSNO into PL/AL hydrogel (Figure 1). First, the alginate solution was prepared at room temperature. Then, pluronic F127 was added to the alginate solution at 4 °C to form PL/AL solution (sol) for further application (final concentrations of PL and AL were 20% and 1% of AL *w*/*v*, respectively). The PL at a concentration of ≥20% can exhibit better thermal reversible behavior [38]. To develop a thermoresponsive gel formulation for local delivery of the NO, the GSNO was incorporated into PL/AL sol at 4 °C, forming a uniform pink GSNO-PL/AL hydrogel. Owing to the presence of PL in the hydrogels, the aqueous GSNO-PL/AL sol was fluid at 4 °C; the viscosity of GSNO-PL/AL sol was increased with increasing temperature, resulting in a decrease in its fluidity at 20 °C; when the temperature increased to 37 °C (body temperature), the GSNO-PL/AL sol transformed to a gel and could not flow again (Figure 1).

After the GSNO-PL/AL hydrogel was synthesized, their physicochemical properties were characterized (Table 1). The gelation temperature (T_gel_) is defined as the temperature required to form the gel phase, which is widely used to characterize the biopolymers that form thermoresponsive gels. The T_gel_ values of PL, PL/AL, and GSNO-PL/AL hydrogels were found to be 26.4 ± 0.2 °C, 24.2 ± 0.3 °C, and 23.4 ± 0.2 °C, respectively (Table 1). The gelation nature of the PL was affected by the addition of AL and GSNO. The AL incorporation on the PL hydrogel decreased the T_gel_ by 2 °C, and the GSNO incorporation on the PL/AL hydrogel slightly decreased the T_gel_ by 1 °C. The addition of drugs and/or co-solvents can considerably change the T_gel_ of the pluronic system [39,40]. The loading of GSNO in the GSNO-PL/AL hydrogel was 1.9%.

**Figure 2:**
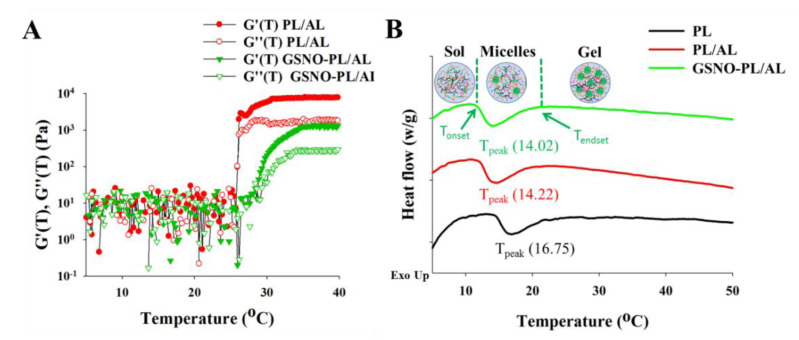
(**A**) Temperature ramp test of PL/AL and GSNO-PL/AL. Changes of storage modulus (G′) and loss modulus (G″) as the temperature changes. (**B**) Differential scanning calorimetry (DSC) thermographs of PL, PL/AL, and GSNO-PL/AL hydrogels.

To evaluate the change of viscoelastic properties of PL/AL and GSNO-PL/AL hydrogels with temperature, a temperature ramp test was performed using ARES. Figure 2A shows the storage modulus (G′) and loss modulus (G″) of PL/AL and GSNO-PL/AL hydrogels as the temperature changes from 5 to 40 °C. In Figure 2A, three distinct stages were observed: solution state at low temperatures (scattering values of G′ and G″), sol−gel transition stages (dramatic changes in G′ and G″), and gel state (higher G′ values than G″). At temperatures below 20 °C, the G′ and G″ values of PL/AL and GSNO-PL/AL remained around 10 Pa and showed scattered patterns, indicating that samples remained in the solution. At the temperature around 20 °C, the G′ and G″ values of the PL/AL and GSNO-PL/AL hydrogels were increased rapidly due to the sol−gel transition (the transition temperatures of the PL/AL and GSNO-PL/AL hydrogels were 24 and 26 °C, respectively). The transition temperature of the GSNO-PL/AL hydrogel was slightly decreased due to the decreased poloxamer contents that resulted from the incorporation of GSNO. At the physiological temperature (around 37 °C), both PL/AL and GSNO-PL/AL showed rheological properties of unflowable hydrogel that could be beneficial as wound dressings.

A DSC profile was used to determine the micellization behavior of the hydrogels (Figure 2B). The micellization process is characterized by the T_onset_, T_peak_, T_endset_, and ∆H. The T_onset_ is the temperature at which micelles begin to form, while T_endset_ is the temperature at which the micellization process is completed. The ∆H is the area under the peak, and the T_peak_ refers to the critical micellization temperature (CMT) [31]. The DSC of the PL (20%) showed a large endothermic peak between T_onset_ = 14.63 °C and T_endset_ = 24.28 °C (Table 1), which is consistent with findings reported in the existing literature [41,42]. The large endothermic peak was ascribed to the micellization of the PL unimers [43]. The DSC thermogram showed a decrease in the CMT when the AL was added to a 20% PL solution, and the T_onset_ changed from 14.63 to 12.20 °C. Conversely, the addition of the GSNO into the PL/AL accelerated the micellization process. This is demonstrated by the decrease in the T_onset_, CMT, and T_endset_, as well as the increase in the endothermic peak area.

### 3.2. In Vitro NO Release

**Figure 3:**
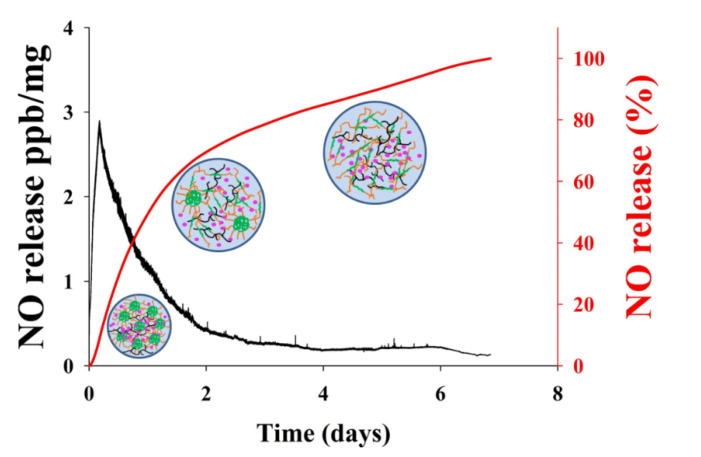
The nitric oxide (NO) release profile from the GSNO-PL/AL hydrogel. The black-colored line shows the real-time monitoring of NO release (ppb/mg) from GSNO-PL/AL hydrogel detected every five seconds; the red-colored line shows the percentage of NO release from the GSNO-PL/AL hydrogel.

NO released in vitro from the GSNO-PL/AL hydrogel at 37 °C was evaluated with the chemiluminescent NO analyzers. The real-time NO release profile and a plot of the percentage of NO released are shown in Figure 3. The real-time NO release graph showed that the maximum NO flux was 2.88 ppb/mg, and it occurred at 4.28 h. In addition, the GSNO-PL/AL hydrogel presented with prolonged NO release over 7 days, and their half-life was ≈24.5 h (Table 2). A total of 8.02% and 28.98% of the NO were released in the first 4 h and 12 h, respectively, followed by a sustained release of 71.2% over 7 days. Previous reports state that the incorporation of GSNO into the hydrogel can decrease the rates of the NO release, with the decrease resulting from the cage effect promoted by the higher viscosity of the hydrogel matrix [44,45]. GSNO-PL/AL hydrogel is a physical gel. After adding the solution to the hydrogel, the surface of the hydrogel will disintegrate layer by layer, due to the hydrogel swelling. So, the NO will quickly release from the hydrogel. As time increases, more and more solution enters the hydrogel. When the concentration of PL in the hydrogel was lower than the critical micelle concentration (CMC), the hydrogel will became a sol at 37 °C. Therefore, it was easy to remove that remaining in the wound bed. The rapid initial NO release in the first 4 h and the subsequent sustained release may eliminate bacterial infections on wounds and promote healing. Therefore, the GSNO-PL/AL hydrogel that facilitates sustained NO release may stimulate healing in MRPA-infected wounds.

### 3.3. In Vitro Cytotoxicity Study

**Figure 4:**
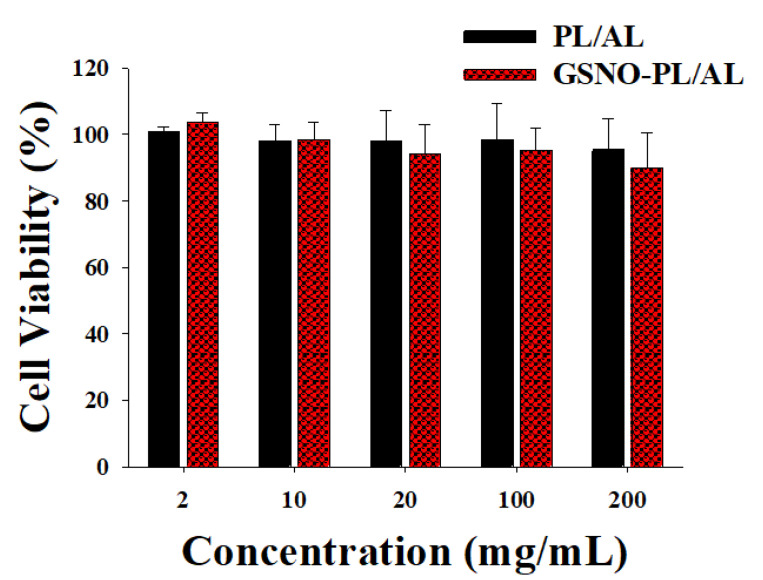
In vitro viability of L929 fibroblast cells after incubating for 24 h with hydrogels at various concentrations. Data shown are mean ± S.D. (n = 6).

The MTT assay was performed to evaluate the cytotoxic effect of the GSNO-PL/AL hydrogel against mammalian cells using the L929 murine fibroblasts. As shown in Figure 4, the PL/AL hydrogels did not show significant cytotoxicity (over 90% of cell viability) up to 200 mg/mL, due to the biocompatible nature of PL and AL. An injectable PL scaffold has been used for tissue engineering. The viability of the L929 after the encapsulation process increased up to 106% on the 5th day of encapsulation [32]. The GSNO-PL/AL also exhibited lower cytotoxic effects, thereby demonstrating no evident cytotoxicity of the GSNO inside the GSNO-PL/AL of the L929 cells. As shown in our previous reports, GSNO was cytotoxic to L929 cells [18]. The inhibition of GSNO cytotoxicity in GSNO-PL/AL was due to the controlled release of NO. As illustrated in Figure 3, GSNO-PL/AL releases NO in a sustained manner over 7 days, thus protecting the cells from exposure to a high amount of NO, thereby implying that NO released from GSNO-PL/AL is safe for topical application. The results indicated that the composite hydrogel was not cytotoxic when applied in wound healing.

### 3.4. In Vitro Antibacterial Activity of GSNO-PL/AL Hydrogel

**Figure 5:**
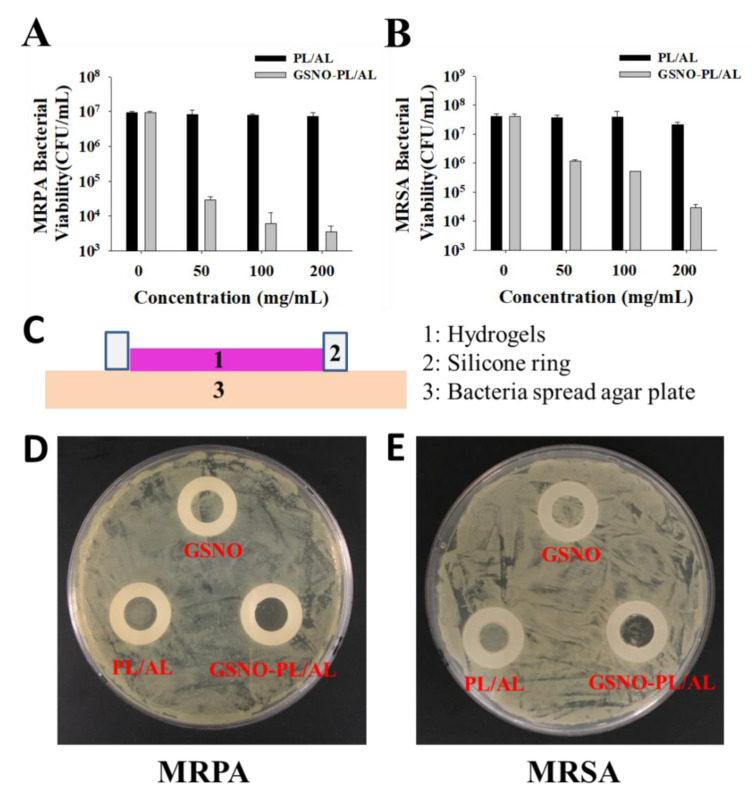
In vitro antibacterial activities of PL/AL and GSNO-PL/AL hydrogels against (**A**) multidrug-resistant *Pseudomonas aeruginosa* (MRPA) and (**B**) methicillin-resistant *Staphylococcus aureus* (MRSA). (**C**) Side-view schematic of antimicrobial study configuration using agar plate with bacterial growth. Macroscopic images of antibacterial activities on the agar plate against (**D**) MRPA and (**E**) MRSA.

NO is well known as a potent antimicrobial agent. We assessed the in vitro antibacterial activity of PL/AL and GSNO-PL/AL hydrogels against Gram-positive (MRSA) and Gram-negative (MRPA) bacteria according to the CFU counts. The GSNO-PL/AL hydrogel at different concentrations (0, 50, 100, and 200 mg/mL) was used to test the antibacterial activity. As shown in Figure 5A,B, PL/AL did not show any antibacterial activity against MRSA or MRPA. In contrast, the GSNO-PL/AL hydrogel showed significant concentration-dependent antibacterial activity. At 200 mg/mL, the GSNO-PL/AL hydrogel caused 3-log and 4-log reductions in MRSA and MRPA viability, respectively. At 100 μg/mL, the GSNO-PL/AL hydrogel caused 2-log and 4-log reductions in the viability of MRSA and MRPA, respectively, while at 50 μg/mL, it caused 1-log and 3-log reductions in the viability of MRSA and MRPA, respectively. Pure PL and AL have no antibacterial effects, but when mixed with GSNO, the hydrogel exhibits an antibacterial effect due to the NO released from the GSNO. The bactericidal properties of NO are attributed to the formation of dinitrogen trioxide (N_2_O_3_) and peroxynitrite (ONOO−), which are the reactive by-products of nitrosative and oxidative stress. These molecules are able to disrupt bacterial membranes, react with intercellular proteins and DNA, and perturb crucial cellular processes, resulting in bacterial cell death [46,47].

When the hydrogel was applied to an infected wound, it was covered on the wound bed. Therefore, to simulate the in vivo antibacterial conditions of the hydrogel, several antibacterial studies were performed with the modified Oxford cup method, as depicted in Figure 5C. The silicone O-ring (inner diameter, 10 mm) was used to form a 10-mm in vitro agar wound model, whereas the inoculated agar plate was used as an infected wound bed. However, because our GSNO-PL/AL hydrogel was solid at 37 °C and the NO released from the GSNO had a short half-life (3–4 s), it was unable to form an evident bacteriostatic zone to show antibacterial properties. However, the GSNO-PL/AL hydrogel in the silicone O-ring area had an inhibitory effect on bacteria. Thus, based on the growth of the bacterial colonies in this area, its antibacterial effect could be qualitatively evaluated. As shown in Figure 5C,D, the GSNO solution and the PL/AL hydrogel had no antibacterial effect, and the growth of MRSA and MRPA in the silicone O-ring was the same outside the circle and was not inhibited. However, after treatment with the GSNO-PL/AL hydrogel, there was no increase in colonies. Thus, these results suggest that the GSNO-PL/AL hydrogel has potent antibacterial activity against MRSA and MRPA. Based on the results, the hydrogels (30 μL) were used for in vivo wound healing.

### 3.5. In Vivo Wound Healing Activity

**Figure 6:**
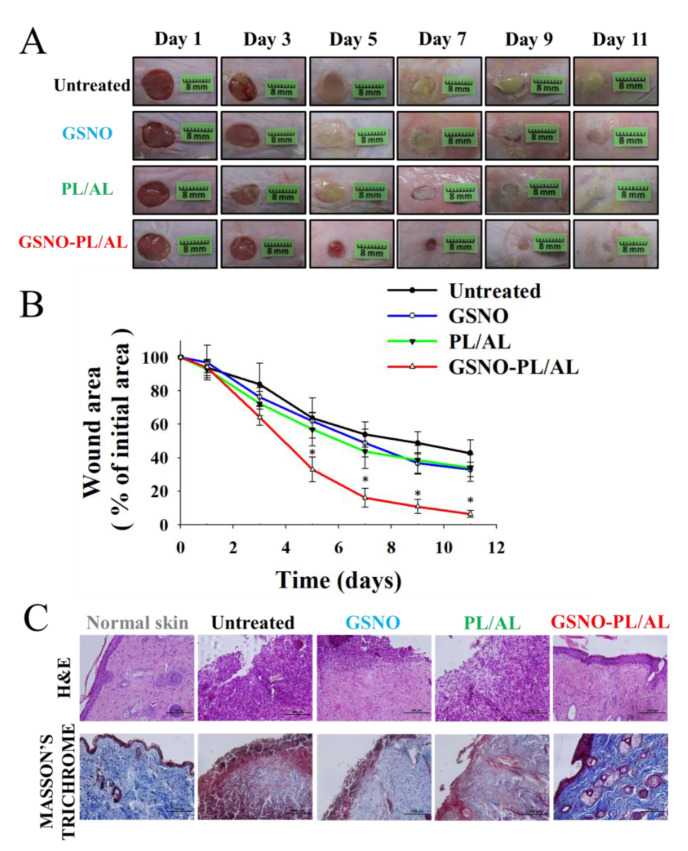
(**A**) Representative photographs of healing burn wounds infected with MRPA in mice treated with PL/AL and GSNO-PL/AL. (**B**) Area reduction (%) profiles of MRPA-infected wounds. Data are presented as means ± SD; n = 6. * *p* < 0.05 vs. untreated. (**C**) Histological analysis (hematoxylin and eosin (H & E) and Masson’s trichrome staining) of MRPA-infected wound at 11 days after injury. Bar = 100 µm.

The in vivo wound-healing activity was assessed to evaluate the healing effects of the prepared hydrogels, and the results have been presented in Figure 6A. The result shows the macroscopic appearance of the wounds on days 1, 3, 5, 7, 9, and 11 post-surgery for untreated, GSNO, and PL/AL hydrogel, without and with GSNO. A reduction in the wound area and an improved re-epithelization was observed in all groups, but these effects were more pronounced in the GSNO-PL/AL group. The untreated, GSNO, and PL/AL hydrogel groups had mild infection and inflammation, which halted the wound treatment on day 11 after injury. By adding GSNO to the hydrogel, wound healing was improved. Wound healing in the GSNO-PL/AL hydrogel-treated group was almost complete at the time.

The percentage of wound area was determined to quantify the wound-healing process (Figure 6B). The wound-healing percentage was the estimated ratio of the recovered wound area to the induced wound area. One day after surgery, the percentage of the initial wound area did not present differences among the groups. The percentage of the wound area in the GSNO, PL/AL hydrogel, and the untreated groups 5 days after surgery was 61.9% ± 5.1%, 56.9% ± 9.8%, and 63.7% ± 12.1%, respectively, and it was 32.9 ± 4.4%, 33.9 ± 8.1%, and 42.8 ± 7.8%, respectively, 11 days after surgery. By adding GSNO, the GSNO-PL/AL treatment resulted in a significant reduction in the wound size compared with the GSNO, PL/AL treatment, and untreated groups. The percentage of the wound area observed on days 5 and 11 post-injury was 32.9 ± 7.4% and 6.4 ± 2.0%, respectively. This result indicated that the GSNO-PL/AL-enhanced wound-healing activity could be attributed to the action of NO released from GSNO in the GSNO-PL/AL hydrogel.

Histological analysis of the skin wounds was performed by H & E and the Masson’s trichrome staining, as shown in Figure 6C. Images of the H & E-stained sections of the GSNO-PL/AL group displayed a morphology that was more similar to that of the healthy skin than the other groups, including minimal hypertrophic scarring, thin epidermis, and nearly normal hair growth on the wound surface. However, the GSNO, PL/AL, and untreated groups showed a large number of monocytes and granulocytes. The Masson’s trichrome staining was performed to evaluate collagen deposition in the wound dermis. Collagen deposits were found in the dermis of the GSNO-PL/AL group, and its morphology resembled that of the normal dermal tissue (Figure 6C). Only weak blue areas were observed in the untreated, GSNO, and the PL/AL groups. Hence, NO release from the GSNO-PL/AL hydrogel plays an essential role in the wound-healing process, such as re-epithelialization and collagen deposition, and it may effectively promote wound healing.

### 3.6. Reduction of Bacterial Burden in Wounds

**Figure 7:**
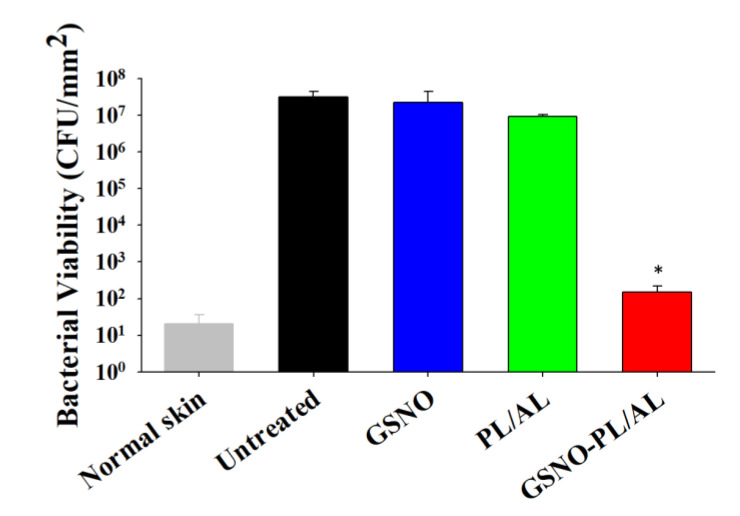
Bacterial burden in wounds at 11 days after injury. * *p* < 0.05 vs. untreated.

We evaluated the reduction in bacterial burden in wounds based on CFU counts. After treatment with free GSNO solution, no antibacterial activity was observed. Figure 7 shows that the PL/AL-treated group exhibited ≈1 log reduction in bacterial viability. However, an insignificant bacterial load was detected in the GSNO-PL/AL group. This finding was consistent with the results of the in vitro bactericidal activity assay.

## 4. Conclusions

In this study, we successfully prepared an NO-releasing thermoresponsive hydrogel (GSNO-PL/AL) that became a pink hydrogel under physiological conditions. The GSNO-PL/AL provided sustained NO release for 7 days and exhibited a rather low cytotoxicity to the L929 mouse fibroblast cells. The GSNO-PL/AL exhibited the most significant bactericidal activity against Gram-positive MRSA and Gram-negative MRPA. Moreover, the GSNO-PL/AL treatment of MRPA-infected wounds accelerated healing and reduced the bacterial burden. The GSNO-PL/AL presented here may be an effective NO-based therapeutic agent against bacterial infections and may promote wound healing.

## Figures and Tables

**Table 1:** Characterization of the hydrogels.

Hydrogels	T_onset_ (°C)	T_peak_ (°C)	T_endset_ (°C)	∆H (j·g^−1^)	T_gel_ (°C)	Loading (%)
PL	14.63	16.75	24.28	2.441	26.4 ± 0.2	N.D.
PL/AL	12.20	14.22	21.87	2.902	24.2 ± 0.3	N.D.
GSNO-PL/AL	11.76	14.02	21.21	3.042	23.4 ± 0.2	1.9 ± 0.2

Data are presented as means ± SD (n = 3). N.D., no data.

**Table 2:** NO release properties of GSNO-PL/AL hydrogel (37 °C).

Material	[NO]_max_ (ppb/mg)	t[NO]_max_ (h)	t_1/2_ (h)	t_d_ (days)
GSNO-PL/AL	2.88	5.82	24.51	7.12

[NO]_max_: maximum NO flux, t[NO]_max_: time to reach [NO]_max_, t_1/2_: the half-life of NO release, t_d_: duration time (time point when 99% of NO is released).

## References

[1] Scatena R., Bottoni P., Pontoglio A., Giardina B. (2010). Pharmacological modulation of nitric oxide release: New pharmacological perspectives, potential benefits and risks. Curr. Med. Chem..

[2] Fang F.C. (1997). Perspectives series: Host/pathogen interactions. Mechanisms of nitric oxide-related antimicrobial activity. J. Clin. Investig..

[3] Cooke J.P. (2003). NO and angiogenesis. Atheroscler. Suppl..

[4] Chen A.F. (2005). Nitric oxide: A newly discovered function on wound healing. Acta Pharmacol. Sin..

[5] Ignarro L.J. (2000). Nitric Oxide: Biology and Pathobiology.

[6] Hill B.G., Dranka B.P., Bailey S.M., Lancaster J.R., Darley-Usmar V.M. (2010). What part of NO don’t you understand? Some answers to the cardinal questions in nitric oxide biology. J. Biol. Chem..

[7] Fang F.C. (2004). Antimicrobial reactive oxygen and nitrogen species: Concepts and controversies. Nat. Rev. Microbiol..

[8] Efron D.T., Most D., Barbul A. (2000). Role of nitric oxide in wound healing. Curr. Opin. Clin. Nutr. Metab. Care.

[9] Hasan N., Cao J., Lee J., Naeem M., Hlaing S.P., Kim J., Jung Y., Lee B.-L., Yoo J.-W. (2019). PEI/NONOates-doped PLGA nanoparticles for eradicating methicillin-resistant *Staphylococcus aureus* biofilm in diabetic wounds via binding to the biofilm matrix. Mater. Sci. Eng. C.

[10] Nurhasni H., Cao J., Choi M., Kim I., Lee B.L., Jung Y., Yoo J.-W. (2015). Nitric oxide-releasing poly (lactic-co-glycolic acid)-polyethylenimine nanoparticles for prolonged nitric oxide release, antibacterial efficacy, and in vivo wound healing activity. Int. J. Nanomed..

[11] Miller M., Megson I. (2007). Recent developments in nitric oxide donor drugs. Br. J. Pharmacol..

[12] Wang P.G., Cai T.B., Taniguchi N. (2005). Nitric Oxide Donors: For. Pharmaceutical and Biological Applications.

[13] Forman H.J. (2016). Glutathione—From antioxidant to post-translational modifier. Arch. Biochem. Biophys..

[14] Hornyák I., Marosi K., Kiss L., Gróf P., Lacza Z. (2012). Increased stability of S-nitrosothiol solutions via pH modulations. Free Radic. Res..

[15] Seabra A.B., De Oliveira M.G. (2004). Poly (vinyl alcohol) and poly (vinyl pyrrolidone) blended films for local nitric oxide release. Biomaterials.

[16] Lee J., Hlaing S.P., Cao J., Hasan N., Yoo J.-W. (2020). In vitro and in vivo evaluation of a novel nitric oxide-releasing ointment for the treatment of methicillin-resistant *Staphylococcus aureus*-infected wounds. J. Pharm. Investig..

[17] Choi M., Hasan N., Cao J., Lee J., Hlaing S.P., Yoo J.-W. (2020). Chitosan-based nitric oxide-releasing dressing for anti-biofilm and in vivo healing activities in MRSA biofilm-infected wounds. Int. J. Biol. Macromol..

[18] Hlaing S.P., Kim J., Lee J., Hasan N., Cao J., Naeem M., Lee E.H., Shin J.H., Jung Y., Lee B.-L. (2018). S-Nitrosoglutathione loaded poly (lactic-co-glycolic acid) microparticles for prolonged nitric oxide release and enhanced healing of methicillin-resistant *Staphylococcus aureus*-infected wounds. Eur. J. Pharm. Biopharm..

[19] Lee J., Hlaing S.P., Cao J., Hasan N., Ahn H.-J., Song K.-W., Yoo J.-W. (2019). In Situ Hydrogel-Forming/Nitric Oxide-Releasing Wound Dressing for Enhanced Antibacterial Activity and Healing in Mice with Infected Wounds. Pharmaceutics.

[20] Lee J., Kwak D., Kim H., Kim J., Hlaing S.P., Hasan N., Cao J., Yoo J.-W. (2020). Nitric Oxide-Releasing S-Nitrosoglutathione-Conjugated Poly (Lactic-Co-Glycolic Acid) Nanoparticles for the Treatment of MRSA-Infected Cutaneous Wounds. Pharmaceutics.

[21] Field C.K., Kerstein M.D. (1994). Overview of wound healing in a moist environment. Am. J. Surg..

[22] Korting H., Schöllmann C., White R. (2010). Management of minor acute cutaneous wounds: Importance of wound healing in a moist environment. J. Eur. Acad. Dermatol. Venereol..

[23] Ousey K., Cutting K., Rogers A.A., Rippon M.G. (2016). The importance of hydration in wound healing: Reinvigorating the clinical perspective. J. Wound Care.

[24] Broussard K.C., Powers J.G. (2013). Wound dressings: Selecting the most appropriate type. Am. J. Clin. Dermatol..

[25] Jones V., Grey J.E., Harding K.G. (2006). Wound dressings. BMJ.

[26] Salehi M., Ehterami A., Farzamfar S., Vaez A., Ebrahimi-Barough S. (2020). Accelerating healing of excisional wound with alginate hydrogel containing naringenin in rat model. Drug Deliv. Transl. Res..

[27] Khodaverdi E., Maftouhian S., Aliabadi A., Hassanzadeh-Khayyat M., Mohammadpour F., Khameneh B., Hadizadeh F. (2019). Casein-based hydrogel carrying insulin: Preparation, in vitro evaluation and in vivo assessment. J. Pharm. Investig..

[28] Perinelli D.R., Cespi M., Bonacucina G., Palmieri G.F. (2019). PEGylated polylactide (PLA) and poly (lactic-co-glycolic acid)(PLGA) copolymers for the design of drug delivery systems. J. Pharm. Investig..

[29] Gou M., Li X., Dai M., Gong C., Wang X., Xie Y., Deng H., Chen L., Zhao X., Qian Z. (2008). A novel injectable local hydrophobic drug delivery system: Biodegradable nanoparticles in thermo-sensitive hydrogel. Int. J. Pharm..

[30] Wagh V.D., Inamdar B., Samanta M. (2008). Polymers used in ocular dosage form and drug delivery systems. Asian J. Pharm..

[31] Al Khateb K., Ozhmukhametova E.K., Mussin M.N., Seilkhanov S.K., Rakhypbekov T.K., Lau W.M., Khutoryanskiy V.V. (2016). In situ gelling systems based on Pluronic F127/Pluronic F68 formulations for ocular drug delivery. Int. J. Pharm..

[32] Yap L.-S., Yang M.-C. (2016). Evaluation of hydrogel composing of Pluronic F127 and carboxymethyl hexanoyl chitosan as injectable scaffold for tissue engineering applications. Colloids Surf. B Biointerfaces.

[33] Abdi S.I.H., Choi J.Y., Lee J.S., Lim H.J., Lee C., Kim J., Chung H.Y., Lim J.O. (2012). In vivo study of a blended hydrogel composed of pluronic F-127-alginate-hyaluronic acid for its cell injection application. Tissue Eng. Regen. Med..

[34] Patole V.C., Pandit A.P. (2017). Mesalamine-loaded alginate microspheres filled in enteric coated HPMC capsules for local treatment of ulcerative colitis: In vitro and in vivo characterization. J. Pharm. Investig..

[35] Pelegrino M.T., Lima B.d.A., Do Nascimento M.H., Lombello C.B., Brocchi M., Seabra A.B. (2018). Biocompatible and Antibacterial Nitric Oxide-Releasing Pluronic F-127/Chitosan Hydrogel for Topical Applications. Polymers.

[36] Tirnaksiz F., Robinson J. (2005). Rheological, mucoadhesive and release properties of pluronic F-127 gel and pluronic F-127/polycarbophil mixed gel systems. Die Pharm..

[37] Yergoz F., Hastar N., Cimenci C.E., Ozkan A.D., Tekinay T., Guler M.O., Tekinay A.B. (2017). Heparin mimetic peptide nanofiber gel promotes regeneration of full thickness burn injury. Biomaterials.

[38] Talasaz A.H., Ghahremankhani A.A., Moghadam S.H., Malekshahi M.R., Atyabi F., Dinarvand R. (2008). In situ gel forming systems of poloxamer 407 and hydroxypropyl cellulose or hydroxypropyl methyl cellulose mixtures for controlled delivery of vancomycin. J. Appl. Polym. Sci..

[39] Dumortier G., Grossiord J.L., Agnely F., Chaumeil J.C. (2006). A review of poloxamer 407 pharmaceutical and pharmacological characteristics. Pharm. Res..

[40] Vercelino R., Cunha T.M., Ferreira E.S., Cunha F.Q., Ferreira S.H., de Oliveira M.G. (2013). Skin vasodilation and analgesic effect of a topical nitric oxide-releasing hydrogel. J. Mater. Sci. Mater. Electron..

[41] Pradines B., Djabourov M., Vauthier C., Loiseau P.M., Ponchel G., Bouchemal K. (2015). Gelation and micellization behaviors of pluronic^®^ F127 hydrogel containing poly (isobutylcyanoacrylate) nanoparticles specifically designed for mucosal application. Colloids Surf. B Biointerfaces.

[42] Zhang M., Djabourov M., Bourgaux C., Bouchemal K. (2013). Nanostructured fluids from pluronic^®^ mixtures. Int. J. Pharm..

[43] Trong L.C.P., Djabourov M., Ponton A. (2008). Mechanisms of micellization and rheology of PEO–PPO–PEO triblock copolymers with various architectures. J. Colloid Interface Sci..

[44] Shishido S.M., Seabra A.B., Loh W., de Oliveira M.G. (2003). Thermal and photochemical nitric oxide release from S-nitrosothiols incorporated in Pluronic F127 gel: Potential uses for local and controlled nitric oxide release. Biomaterials.

[45] Picheth G.F., Marini T.C., Taladriz-Blanco P., Shimamoto G.G., Dos Santos G.J., Meneau F., de Oliveira M.G. (2019). Influence of Pluronic F127 microenvironments on the photochemical nitric oxide release from S-nitrosoglutathione. J. Colloid Interface Sci..

[46] Burgner D., Rockett K., Kwiatkowski D. (1999). Nitric oxide and infectious diseases. Arch. Dis. Child..

[47] Lu Y., Slomberg D.L., Schoenfisch M.H. (2014). Nitric oxide-releasing chitosan oligosaccharides as antibacterial agents. Biomaterials.

